# Supplementary material for: Control of morphology and formation of highly geometrically confined magnetic skyrmions
Source: Nat Commun. 2017 Jun 5;8:15569. doi: 10.1038/ncomms15569 (PMC5465359; doi:10.1038/ncomms15569)
Supplement: Supplementary Information — Supplementary figures and supplementary note. [file ncomms15569-s1.pdf]

Supplementary Information

Supplementary Figures

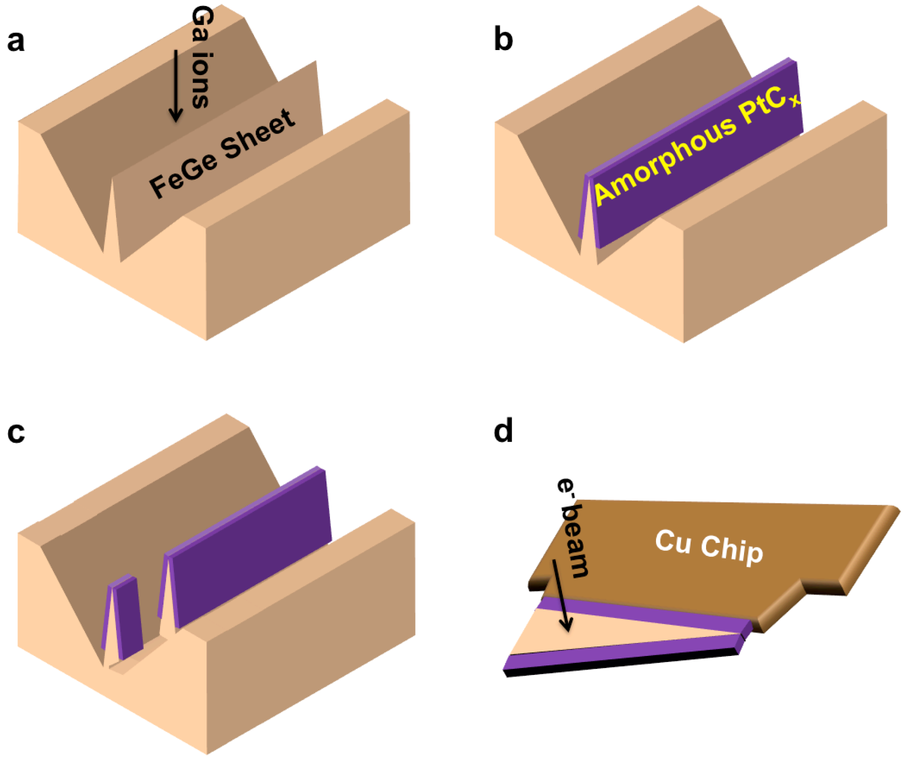

Supplementary Fig. 1 | Procedure used to prepare a wedge-shaped FeGe nanostripe. The following four steps were carried out in a FIB-SEM dual-beam workstation (FEI Helios NanoLab 600i) equipped with a gas injection system (GIS) and an Omniprobe 200 micromanipulator: **a**, A Ga ion beam (indicated by the arrow) was used to FIB mill a thin FeGe sheet on the surface of a bulk FeGe specimen. By controlling the tilt angle of the specimen, the FeGe sheet could be fabricated with a variable thickness profile. No protective PtC_X layers were used during this step, resulting in the possibility of damage to the very thin edge by Ga ion bombarment. **b**, By using the GIS system, a layer of amorphous PtC_X was deposited onto the FeGe sheet using electron-beam-assisted chemical vapor deposition, in order to prevent Ga ion beam damage during the subsequent steps. The PtC_X layer also subsequently reduced the influence of Fresnel fringes at the edges of the FeGe in Lorentz TEM images. **c**, A slot was cut in the FeGe sheet using FIB milling with Ga ions, in order to seperate the desired region from the bulk specimen. **d**, The final electron-transparent FeGe nanostripe, still encapsulated in amorphous PtC_X, was attached to a Cu TEM chip using the Omniprobe micromanipulator. The arrow indicates the direction of the electron beam impinging on the specimen in the transmission electron microscope.

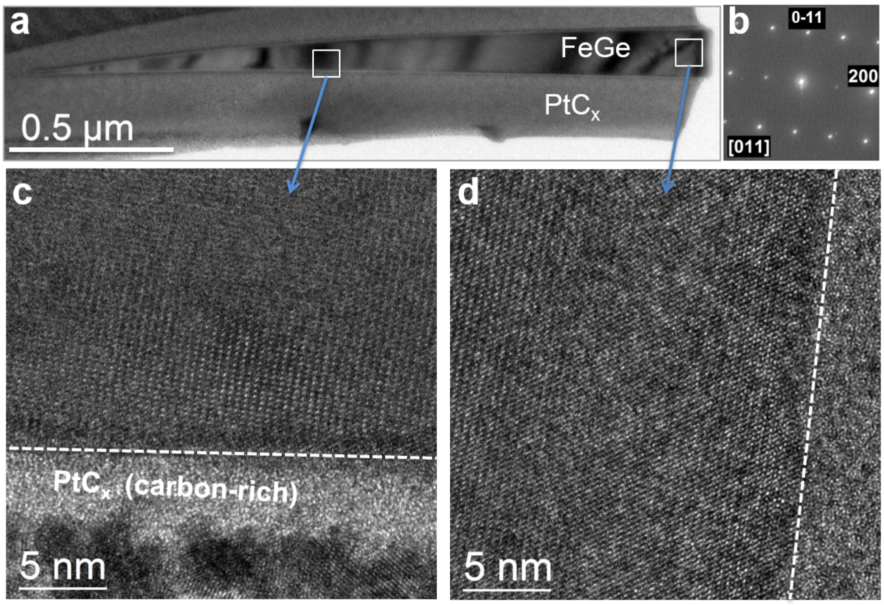

Supplementary Fig. 2 | Morphology and crystal structure of the FeGe nanostripe. **a**, Bright-field TEM image. **b**, Electron diffraction pattern with annotations showing the crystallographic orientation. **c** and **d**, High-resolution TEM images recorded from the areas marked by the white squares in **a**. The images show the crystalline nature of the specimen to its very edge. The TEM images and electron diffraction pattern are consistent with the B20 cubic crystal structure of FeGe.

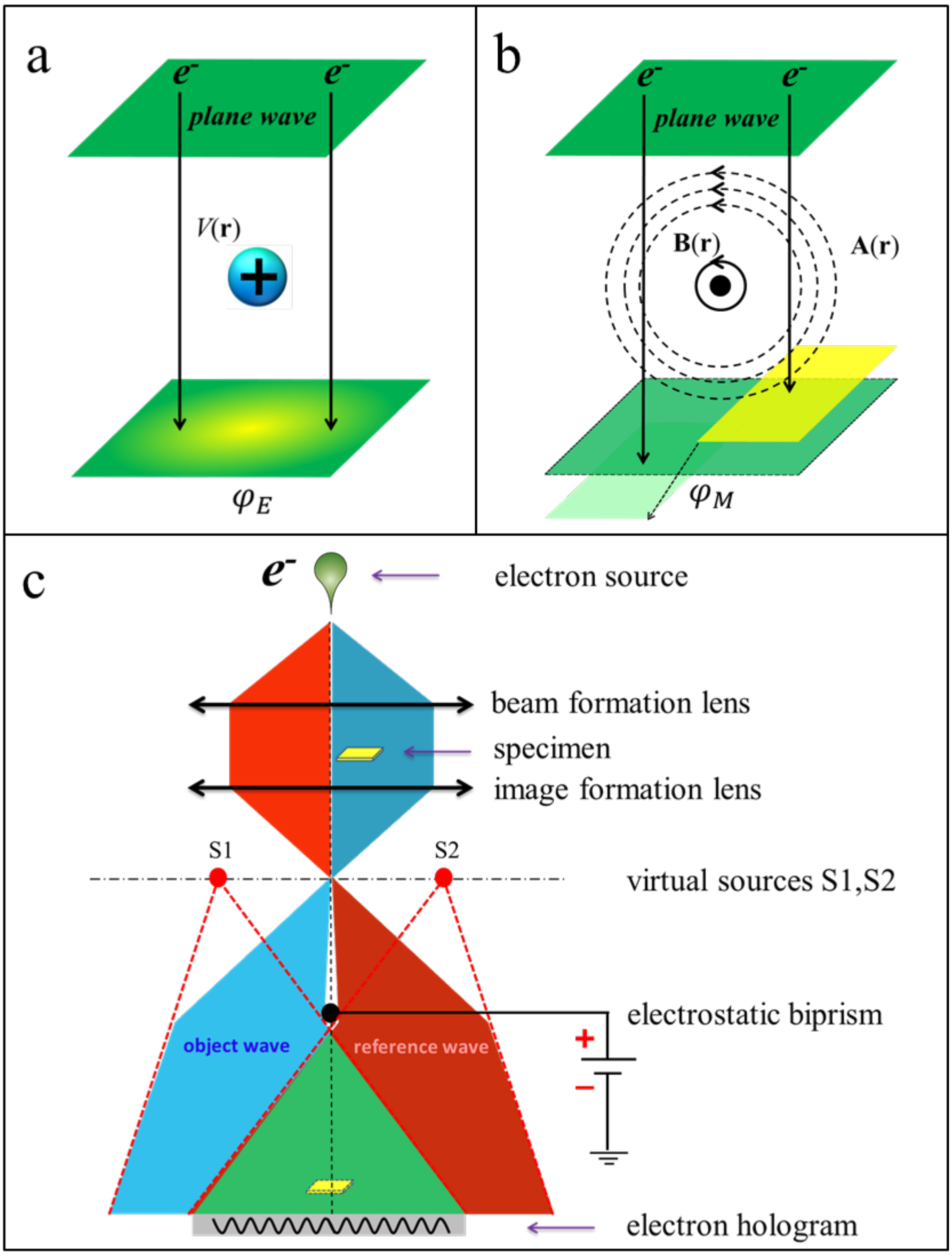

Supplementary Fig. 3 | Contributions to the electron holographic phase shift. a, Electrostatic phase contribution φ_E originating from the electrostatic potential $V(\mathbf{r})$ within and around the TEM specimen. **b**, Magnetic phase contribution φ_M originating from the magnetic vector potential **A(r)** within and around the TEM specimen. **c**, Simplified ray diagram for off-axis electron holography. Essential components are the coherent electron source, electromagnetic lenses and electrostatic biprism for separation and overlap of two parts of the electron wave to form an electron hologram. The object and reference waves can be considered as originating from two virtual sources S1 and S2. The electron-transparent specimen occupies approximately half of the field of view. For recording information about the magnetic properties of the specimen, the conventional TEM objective lens is normally switched off and a non-immersion Lorentz lens is used as the primary imaging lens. A pre-calibrated magnetic field can then be applied to the specimen in the electron beam direction by exciting the conventional objective lens slightly. The final electron hologram can be recorded digitally for further analysis to yield information about the projected electromagnetic potential within and around the specimen.

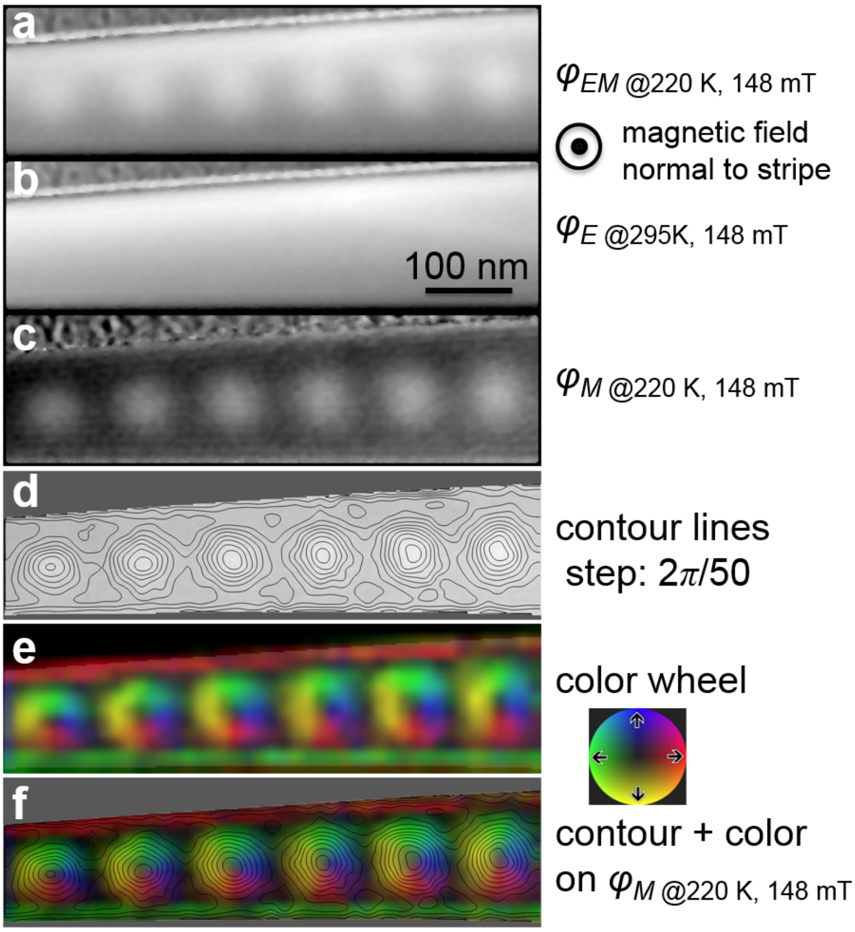

Supplementary Fig. 4 | Separation of contributions to a phase image. a, Original (total) phase image φ_{EM} recorded at 220 K in an applied magnetic field of 148 mT. **b**, Electrostatic contribution to the phase φ_E recorded at 295 K in an applied magnetic field of 148mT. **c**, Magnetic contribution to the phase φ_M obtained by subtracting φ_E from φ_{EM}. **d**, Magnetic phase contour lines (spacing $2\pi/50$ radians) determined from φ_M in **c** showing the projected in-plane magnetic induction. **e**, Color map determined from the gradient of φ_M in **c**, representing the direction and magnitude of the projected in-plane magnetic induction in its hue and brightness, respectively, according to the color wheel shown on the right (red = right, yellow = down, green = left, blue = up). **f**, Magnetic induction map shown in the form of a composite color-contour image. See the Supplementary Note below for further details.

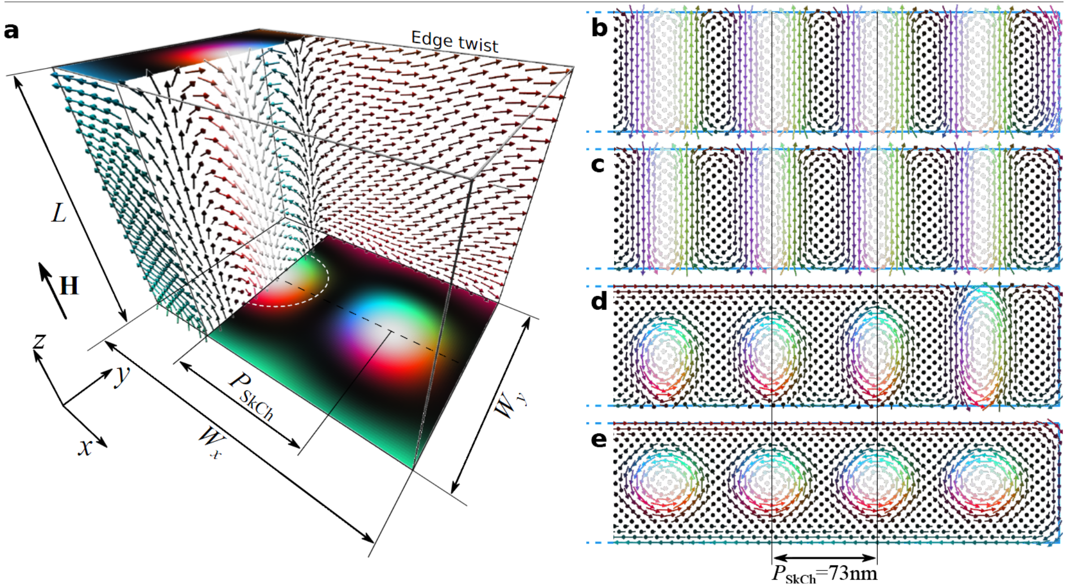

Supplementary Fig. 5 | Evolution of spin structure in a confined geometry. a, Geometry of a simulated domain used to calculate the phase diagram shown in Fig. 4b in the main text. The thickness L in all of the calculations is fixed to 110 spins, while W_y is varied between 30 and 240 spins and W_x is usually adapted to fit the equilibrium period of the skyrmion chain P_{SkCh}. Periodic boundary conditions are applied along the x-axis. The magnetization distribution in the xz and yz cutting planes illustrates the spin structure inside the skyrmion tube and the effect of magnetization twist at the free edge of the specimen. The color map in the xy cutting plane illustrates the magnetization distribution according to the color sphere shown in the inset to Fig. 3c in the main text. **b-e** illustrate the evolution of spin structure in the middle plane of the simulated domain as a function of applied magnetic field (**b**, 0.0 mT; **c**, 0.1 mT; **d**, 0.13 mT; **e**, 0.13 mT) for the case W_y = 90 nm. Here, open boundary conditions are applied in all three spatial directions. The blue line indicates the free edges of the specimen. Only the side that is close to the right edge of the simulated domain is shown. **d** shows incomplete skyrmion states during the skyrmion chain formation from the helical phase, while the fully relaxed spin structure is shown in **e**. The periods of the initial equilibrium spin spiral and the final single skyrmion chain are equivalent, while the equilibrium value of P_{SkCh} corresponding to an energy minimum is ~85 nm. Only ~1:10 of the spins are shown in each figure for ease of visual interpretation.

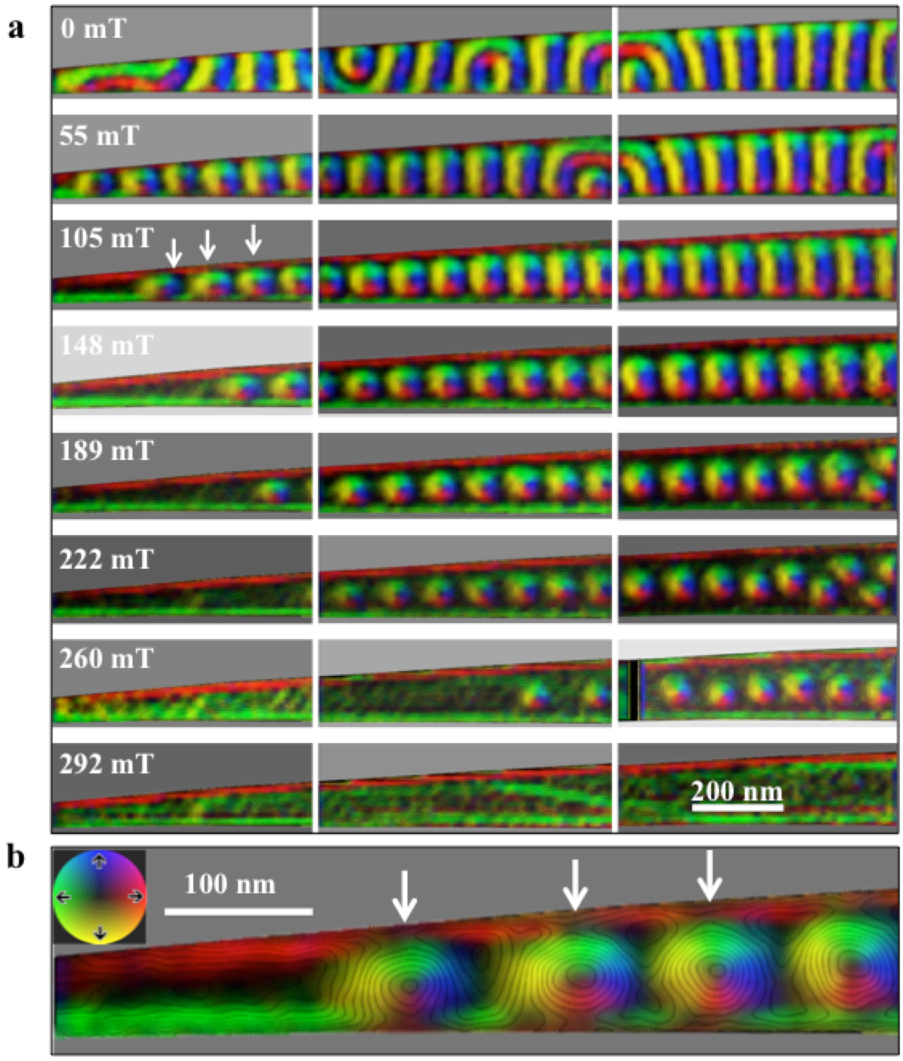

Supplementary Fig. 6 | Magnetic induction maps recorded at 220 K. a, Magnetic induction maps showing a cascade of magnetic transitions through spiral, elongated skyrmion, circular skyrmion and field-induced ferromagnetic states. In order to record the magnetic microstructure of the nanostripe with high spatial resolution, off-axis electron holograms of three different parts of the nanostripe were recorded separately. The images shown in Fig. 2 in the main text were created by stitching together three individual magnetic induction maps to obtain a larger field of view. **b**, Enlargement of an intermediate state at 105 mT (marked by white arrows in **a**), which is characterized by half-skyrmions at one edge and a twisted edge state at the other edge. The magnetic induction map in **b** illustrates some of the different magnetic structures that were observed when different magnetic fields were applied to the nanostripe.

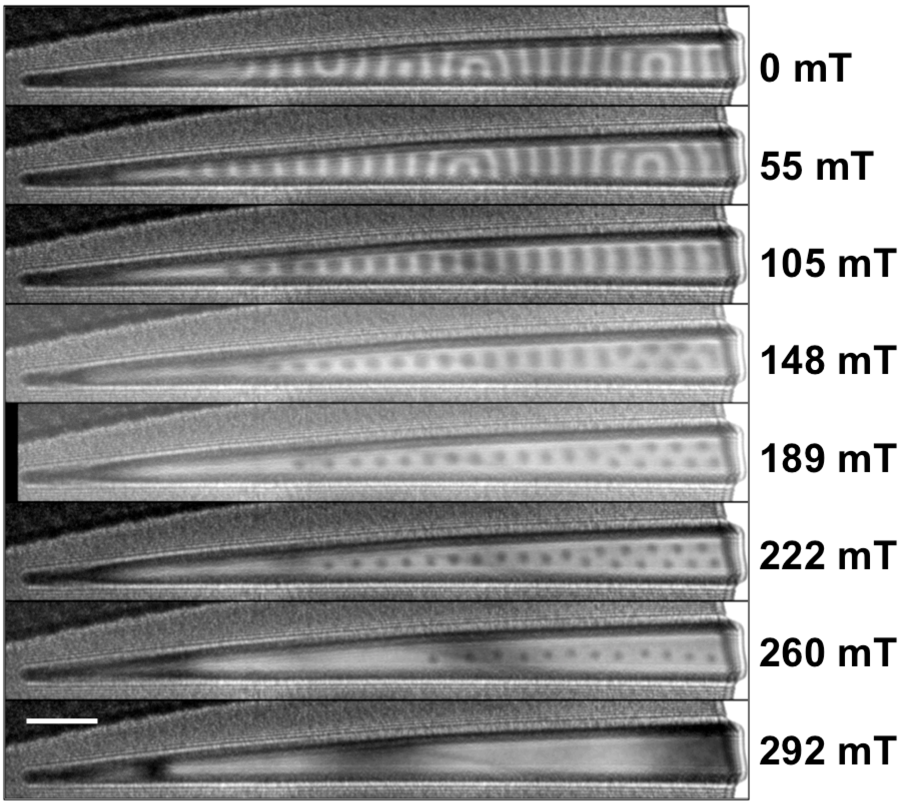

Supplementary Fig. 7 | Lorentz TEM images recorded at 220 K. The images were used together with the electron holography results to construct a *W-H* phase diagram from a wider region of the specimen. It should be noted that accurate magnetic imaging using Lorentz TEM is extremely difficult for specimens with very small feature sizes (tens of nm and below) because Fresnel fringe contrast associated with changes in specimen thickness and composition at material edges and boundaries can dominate the magnetic signal of primary interest. This difficulty is apparent at the edges of the FeGe nanostripe, where Fresnel fringes associated with the change in specimen thickness and composition dominate the contrast. The magnetic signal close to such features is easier to separate from phase images recorded using off-axis electron holography than from defocused images recorded using Lorentz TEM. The scale bar is 200 nm.

Supplementary Note 1 | Off-axis electron holography for imaging magnetic contrast

According to the Ehrenberg-Siday-Aharonov-Bohm (ESAB) effect in quantum mechanics, the wave function of an electrically charged particle is affected by the electromagnetic potential through which it traverses. In transmission electron microscopy (TEM), the phase change of an electron wave that traverses an electron-transparent specimen (written in one dimension here for simplicity) can be expressed in the form

$$\varphi_{\mathrm{EM}}(\mathrm{x}) = \varphi_{\mathrm{E}} + \varphi_{\mathrm{M}} = C_{\mathrm{E}} \int V(x,z)dz - 2\pi \frac{e}{h} \iint B_{\perp}(x,z)dxdz, \quad (1)$$

where x is a direction in the plane of the specimen, z is the incident electron beam direction, C_{E} is an interaction constant that takes a value of 6.53×10^{6} rad $\cdot$ V $\cdot$ m^{-1} at a microscope accelerating voltage of 300 KV, V is the electrostatic potential within and around the specimen and $B_{\perp}$ is the in-plane component of the magnetic induction within and around the specimen. The total recorded phase φ_{EM} is the sum of the electrostatic contribution to the phase φ_{E} originating from the electrostatic potential $V(\mathbf{r})$ (see Supplementary Fig. 3a) and the magnetic contribution to the phase φ_{M} (see Supplementary Fig. 3b).

One of the most widely used techniques for recording the total phase φ_{EM} within and around a specimen directly is the TEM mode of off-axis electron holography. The technique requires the use of a highly coherent field emission gun (FEG) electron source to examine a specimen, in which the region of interest is positioned so that it occupies approximately half of the field of view. The application of a voltage to an electron biprism results in overlap of part of the electron wave that has passed through vacuum alone with part of the same electron wave that has passed through the specimen, as shown schematically in Supplementary Fig. 3c. If the electron source is sufficiently coherent, then an interference fringe pattern (an electron hologram) is formed in the overlap region, in addition to an image of the specimen. The amplitude and the phase of the specimen wave are encoded in the intensity and the position, respectively, of the interference fringes. For studies of magnetic materials, a Lorentz lens (a high-strength minilens) allows the microscope to be operated at high magnification with the objective lens switched off and the sample located in magnetic-field-free conditions. An external magnetic field can then be applied to the specimen either by using a magnetizing specimen holder or by exciting the conventional microscope objective lens to a pre-calibrated value.

In the present study of the FeGe nanostripe, the total phase change φ_{EM} is a sum of the electrostatic contribution to the phase φ_{E} arising from local variations in specimen thickness and composition and the magnetic contribution to the phase φ_{M} arising from the magnetic vector potential associated with the specimen. Since the magnetic phase information is of primary interest, φ_{M} has to be separated from φ_{E}, especially close to the edge of the FeGe nanostripe, where the specimen thickness and composition change rapidly. Supplementary Fig. 4 shows the experimental procedure that was used here to separate the magnetic contribution to the phase using off-axis electron holography. Figure 4a shows the total recorded phase of a segment of the FeGe nanostripe in an applied magnetic field $\mu_0 H \sim 148$ mT at a temperature $T \sim 220$ K.

The spotty contrast in the center of the nanostripe arises from the formation of individual skyrmions. However, the dominant contrast arising from the electrostatic contribution to the phase φ_{E} hinders interpretation of the detailed skyrmion structure. In order to separate φ_{M} from φ_{E}, we subtracted the electrostatic contribution to the phase φ_{E} (Supplementary Fig. 4b) from the total phase φ_{EM} (Supplementary Fig. 4a) to obtain the pure magnetic phase φ_{M} (Supplementary Fig. 4c). The electrostatic contribution to the phase φ_{E} shown in Supplementary Fig. 4b was recorded at room temperature, well above the Curie temperature ($T_{\mathrm{C}} \sim 280$ K) of FeGe. A magnetic induction map was generated from the resulting magnetic contribution to the phase, both using phase contour lines of spacing $2\pi/50$ radians (Supplementary Fig. 4d) and using colors (Supplementary Fig. 4e) to represent the directions and magnitudes of the measured projected in-plane magnetic induction. Supplementary Fig. 4f shows a final composite contour-color representation. Note that only the projected magnetic induction inside the specimen is shown in the present images, while the very weak fields outside the specimen are masked out. Note also that the presence of a PtC_X layer around the FeGe may have reduced changes in electron-beam-induced charging of the FeGe nanostripe with temperature. Such changes in electron-beam-induced charging with temperature could have resulted in an inability to form reliable magnetic induction maps using the procedure described above.
